# Mitochondrial dysfunction and ischemia in critical illness: an adipose tissue microdialysis study in 203 ICU patients

**DOI:** 10.1186/cc14112

**Published:** 2015-03-16

**Authors:** M Theodorakopoulou, S Apollonatou, N Nikitas, D Vassiliadi, A Diamantakis, V Tsagkari, F Frantzeskaki, I Dimopoulou

**Affiliations:** 1 University Hospital of Athens, Greece

## Introduction

Ischemia and mitochondrial dysfunction have been implicated in critical illness. The potential of MD to diagnose and separate ischemia and mitochondrial dysfunction in ICU patients remains currently unknown.

## Methods

A retrospective, observational study of 203 mechanically ventilated patients studied over a 6-year period with MD including medical, surgical and trauma patients. Sepsis stages: SIRS (*n *= 24), severe sepsis (*n *= 46) and septic shock (*n *= 133). Median age 67 years (range: 17 to 92 years). Mortality was 53%. All subjects had a MD catheter placed in femoral adipose tissue upon admission to the ICU. Interstitial fluid samples were collected six times per day, for 3 consecutive days, and were analyzed for glucose, lactate, pyruvate, and glycerol levels. The lactate to pyruvate (LP) ratio was calculated. Blood lactate was measured. Ischemia was defined as LP ratio >30 and pyruvate level <70 mmol, while mitochondrial dysfunction was defined as LP ratio >30 and pyruvate >70 mmol.

## Results

Analysis during the course of the 3-day period revealed three distinct patterns: no ischemia/mitochondrial dysfunction (*n *= 150 or 74%), ischemia (*n *= 27 or 13%) and mitochondrial dysfunction (*n *= 26 or 13%). On day 1, median blood lactate was higher in mitochondrial dysfunction (2.2 mmol/l) compared with both ischemia (1.3 mmol/l) and with no ischemia/mitochondrial dysfunction (1.3 mmol/l) (*P *= 0.004). Again on day 1, median interstitial fluid lactate was higher in mitochondrial dysfunction (8.4 mmol/l), in comparison with ischemia (1.4 mmol/l) and with the group without ischemia/mitochondrial dysfunction (2.5 mmol/l) (*P *< 0.001). Similar results were obtained with interstitial fluid glycerol levels (*P *= 0.009). Median LP ratio was higher in ischemia (LP = 36), and mitochondrial dysfunction (LP = 33) compared with those without ischemia/mitochondrial dysfunction (LP = 17) (*P *< 0.001). Median interstitial fluid glucose was lower in ischemia (2 mmol/l) compared with both mitochondrial dysfunction (4 mmol/l) and with no ischemia/mitochondrial dysfunction (5 mmol/l) (*P *< 0.001). ICU mortality was 77% in mitochondrial dysfunction, 52% in ischemia and 49% in the group without ischemia/mitochondrial dysfunction (*P *= 0.033).

## Conclusion

Bedside subcutaneous adipose tissue MD is possible to diagnose and separate ischemia and mitochondrial dysfunction in general ICU patients. These two conditions are not so common; however, mitochondrial dysfunction seems to be associated with higher mortality rates.

